# Key Soybean Seedlings Drought-Responsive Genes and Pathways Revealed by Comparative Transcriptome Analyses of Two Cultivars

**DOI:** 10.3390/ijms23052893

**Published:** 2022-03-07

**Authors:** Huidong Xuan, Yanzhong Huang, Li Zhou, Sushuang Deng, Congcong Wang, Jianyu Xu, Haitang Wang, Jinming Zhao, Na Guo, Han Xing

**Affiliations:** National Center for Soybean Improvement, Key Laboratory of Biology and Genetics and Breeding for Soybean, Ministry of Agriculture, State Key Laboratory for Crop Genetics and Germplasm Enhancement, College of Agriculture, Nanjing Agricultural University, Nanjing 210095, China; 2017201035@njau.edu.cn (H.X.); huangyanzhong@163.com (Y.H.); 2019101079@stu.njau.edu.cn (L.Z.); dengsushuang@163.com (S.D.); 2018201069@njau.edu.cn (C.W.); 2019101130@njau.edu.cn (J.X.); 2012093@njau.edu.cn (H.W.); jmz3000@126.com (J.Z.)

**Keywords:** *Glycine max* [L.] Merr., seedling stage, drought stress, RNA-seq, signal transduction pathways

## Abstract

Seedling drought stress is one of the most important constraints affecting soybean yield and quality. To unravel the molecular mechanisms under soybean drought tolerance, we conducted comprehensive comparative transcriptome analyses of drought-tolerant genotype Jindou 21 (JD) and drought-sensitive genotype Tianlong No.1 (N1) seedlings that had been exposed to drought treatment. A total of 6038 and 4112 differentially expressed genes (DEGs) were identified in drought-tolerant JD and drought-sensitive N1, respectively. Subsequent KEGG pathway analyses showed that numerous DEGs in JD are predominately involved in signal transduction pathways, including plant hormone signaling pathway, calcium signaling pathway, and MAPK signaling pathway. Interestingly, JA and BR plant hormone signal transduction pathways were found specifically participating in drought-tolerant JD. Meanwhile, the differentially expressed *CPKs*, *CIPKs*, *MAPKs,* and *MAP3Ks* of calcium and MAPK signaling pathway were only identified in JD. The number of DEGs involved in transcription factors (TFs) is larger in JD than that of in N1. Moreover, some differently expressed transcriptional factor genes were only identified in drought-tolerant JD, including *FAR1*, *RAV*, *LSD1*, *EIL*, and *HB-PHD*. In addition, this study suggested that JD could respond to drought stress by regulating the cell wall remodeling and stress-related protein genes such as *EXPs*, *CALSs*, *CBPs*, *BBXs*, and *RD22s*. JD is more drought tolerant than N1 owing to more DEGs being involved in multiple signal transduction pathways (JA, BR, calcium, MAPK signaling pathway), stress-related TFs, and proteins. The above valuable genes and pathways will deepen the understanding of the molecular mechanisms under drought stress in soybean.

## 1. Introduction

Soybean (*Glycine max* [L.] Merr.) has been adopted as one of the most important economic and oil crops worldwide. It provides human beings with major plant oils, proteins, macronutrients, minerals, and isoflavones [1,2]. However, there are several environmental challenges including biotic and abiotic stresses. Among those, drought stress is gradually increasing with the changes of global climatic conditions [3]. Studies have reported that drought can significantly reduce the yield of soybean by 24–50%, or more seriously by 80% or higher [4,5]. Therefore, efforts need to be made to understand the potential molecular mechanisms and genes which are related to soybean drought tolerance.

Drought stress can induce many adverse effects in plants, such as osmotic imbalance, membrane system damage, and decrease in respiration and photosynthetic rate [6]. To cope with drought stress, plants initiate multiple drought response strategies at the morphological, physiological and molecular levels, such as changing the morphological structure of roots and leaves, promoting the synthesis of hormones and osmotic regulators, and regulating the expression of drought-tolerant genes [7]. Drought stress can change the biosynthesis and signal transduction of various plant hormones, including abscisic acid (ABA), ethylene (ET), auxin (AUX), salicylic acid (SA), jasmonates (JA)and brassinosteroids (BR) [8,9]. Previous studies have shown that endogenous plant hormones play an important role in regulating plant adaption to drought conditions [10]. Several hormonal signal transduction factors related to drought stress have been identified, including abscisic acid responsive elements binding factor (ABF), ethylene insensitive 3/EIN3-like (EIN3/EIL), AUX/IAAand BRASSINOSTEROID INSENSITIVE2 (BIN2) [11,12,13,14]. In addition, some transcription factors (TFs) have been demonstrated to be associated with drought tolerance in plants, including WRKY, MYB, NAC, and DREB [15,16]. Numerous conserved drought response proteins have been identified, such as drought response protein (RD22) and late embryogenesis rich protein (LEA), which increase the water binding capacity of cells under drought stress [17,18]. Although current research on drought has discovered these, plant responses to drought stress are still ambiguous [19]. Therefore, it is a challenge to identify more drought-responsive genes and complex regulatory mechanisms and use the identified useful genes to improve the drought tolerance of plants.

Omics technology, including genomics, transcriptomics, proteomics and metabolomics, have greatly contributed to the understanding of gene and protein functions in plant responses to abiotic stress with their advanced science and technology. With the recent decreased cost of next-generation sequencing, RNA-seq based transcriptome analysis has become one of the most effective strategies to analyze the interactive mechanism between plants and abiotic stress [20]. In rice, specific dependent genes for drought tolerance were identified in drought response mechanism of the comparison between drought-tolerant rice landraces and drought-sensitive modern rice varieties by RNA-seq [21]. Illumina RNA sequencing was used to obtain the gene expression profiles of two maize inbred lines with different drought tolerance levels in response to drought stress at the seedling stage, which found that the different genes in drought tolerance may be related to different ROS scavenging capabilities, signal interaction networks and some TFs [22]. With the release of soybean genome database, transcriptome sequencing technology has been successfully applied to study the expression levels of the key genes in soybean under water-deficit conditions [23]. For example, a microarray study of two soybean genotypes revealed some differentially expressed genes (DEGs) under dehydration and rehydration conditions [24]. Transcriptomic analyses from soybean PI 416937 and the cultivar ‘Benning’ showed that some up-regulated DEGs involved protein transport and chromatin remodeling [25].

Although the mechanisms of drought tolerance in soybean have been studied previously by transcriptome sequencing technology, studies have focused on analyzing the mechanisms of dehydration treatment and drought treatment in post-vegetative growth period (V5-R2) and reproductive growth period [26,27]. Meanwhile, other reports have focused on the analysis of the response mechanism of dehydration treatment or instantaneous osmotic stress treatment with 8% PEG8000 in soybean seedling stage [24,28]. Drought stress is a slow process for plants in the soil, rather than instantaneous [26]. However, drought stress in the seedling stage (pre-vegetative) of plants is a common abiotic stress caused without rainfall or untimely irrigation, which seriously affects the establishment of plant seedlings and leads to considerable yield loss [29]. The soybean seedling stage requires less water than reproductive growth stage, while drought stress at the seedling stage (V2-V3) directly affects the seedling rates and quality of soybeans, and ultimately the soybean yield. So far, there have been few reports on the molecular mechanisms and genetic basis of soybean response to drought treatment at the seedling stage. Above all, it is a crucial task to study the mechanisms of soybean response to drought stress and identify drought-tolerant genes in soybean seedlings stage. Therefore, the present study used RNA-seq approach to investigate transcriptomic profiles of two soybean cultivars in different drought tolerance levels, Jindou 21 (JD, drought-tolerance) and Tianlong No.1 (N1, drought-sensitive), subjected to drought stress at seedling stage, in which candidate genes and molecular mechanisms of drought tolerance were identified at the soybean seedling stage. Our study aims to reveal the molecular mechanisms of drought tolerance between two soybean varieties at the seedling stage from the transcriptional level, which provides a certain research basis for improving the tolerance of soybean seedlings to drought stress in the future.

## 2. Results

### 2.1. Phenotypic Response of Jindou 21 and Tianlong No.1 to Drought Stress

**Figure 1:**
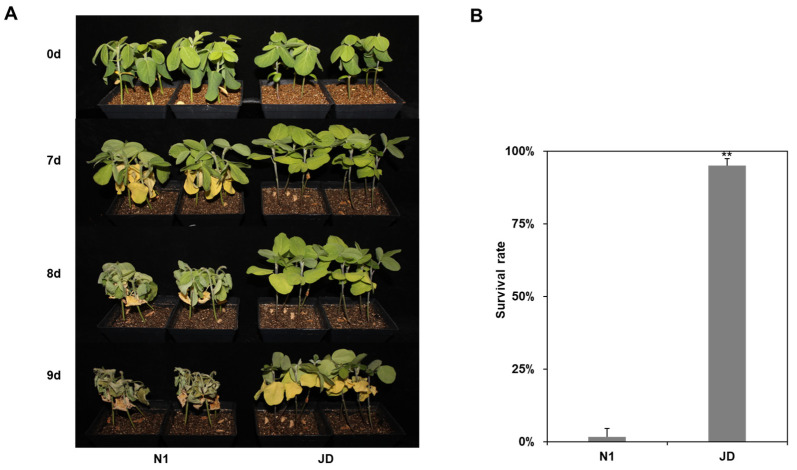
Phenotypic characterization of Tianlong No.1 (N1) and Jindou 21 (JD) under drought stress. (**A**) Phenotypes of N1 and JD on 0 d, 7 d, 8 d and 9 d after drought treatment. (**B**) Statistics of survival rate of N1 and JD after 9 days of drought treatment. Forty seedlings in N1 and JD were used for survival rate. Data represent the means ± SD of three replicates. **, *p* < 0.01.

To investigate the drought tolerance phenotype of soybean JD and N1, 2-week-old seedlings of JD and N1 were selected for drought treatment. Under 7 d of drought treatment, the leaves of N1 turned yellow and wilting while the JD leaves remained in good condition (Figure 1A). At 9 d of drought treatment, the leaves and stems of N1 almost completely dehydrated, while the leaves of JD begun to turn yellow and wilting (Figure 1A). Then, soybean seedlings were rewatered. After 2 d of rewater, the statistics of survival rate showed that JD has a significantly higher survival rate than N1 (Figure 1B). The above results showed that the drought tolerance of JD was better than that of N1 under drought conditions in the seedling stage. Therefore, these two cultivated soybean varieties were used for transcriptome analysis to explore the drought response mechanism of soybean and the mining of related drought tolerance genes at the seedling stage.

### 2.2. Transcriptome Profiles of JD and N1 Soybeans Exposed to Drought Stress

To reveal the molecular mechanism of soybean response to drought stress on the seedling stage, 12 cDNA libraries of the above two soybean genotypes at control and drought stress were constructed and sequenced with three biological replicates. A total of 82.98 G of clean data was obtained using the HiSeq X Ten platform (Illumina). The clean base of each sample was distributed between 6.87–6.94 G, Q30 bases were distributed between 92.94–93.86%, and the average GC content was 45.45% (Table S1). By comparing reads to the reference genome, the genomic alignment of each sample was obtained, and the alignment rate was 96.25–97.50% (Table S2). For global comparison of the transcriptomes derived of two soybean genotypes with three repeats under control and drought conditions, we performed a principal component analysis (PCA). The first two principal components (PC1 and PC2) accounted for 81% of the total variance (Figure S1). Hence, the results indicated that there were different gene expression patterns between the two soybean genotypes under drought stress.

### 2.3. Identification of DEGs of Jindou 21 and Tianlong No.1 to Drought Stress

**Figure 2:**
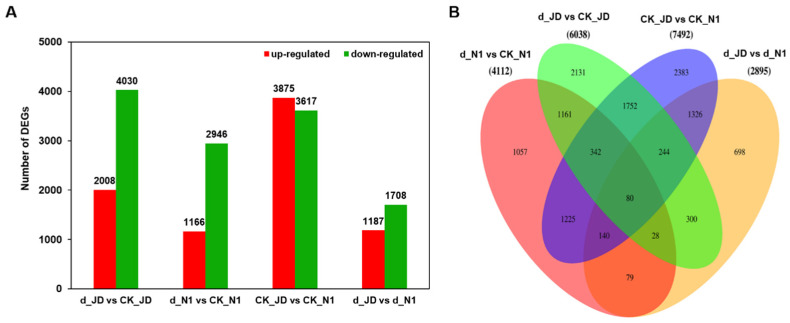
Differentially expressed genes (DEGs) between tested samples. (**A**) Numbers of DEGs compared between two samples DEGs are shown in red (up-regulated) and green (down-regulated). (**B**) Venn diagram of the DEGs in two soybean genotypes under control and drought stress. d_JD means the seedlings of Jindou 21 under the drought treatment; CK_JD means the seedlings of Jindou 21 under the normal; d_N1 means the seedlings of Tianlong No. 1 under the drought treatment; CK_N1 means the seedlings of Jindou 21 under the normal.

Drought stress induced expression changes of a large number of genes in plants. The number of these DEGs (|log_2_FoldChange| > 1, *p* < 0.05) in the two drought-stressed soybean genotypes is shown in Figure 2A. A total of 6038 DEGs were found in the drought-stressed JD, in which 2008 genes were up-regulated and 4030 were down-regulated. In contrast, 4112 DEGs were generated with 1166 up-regulated and 2946 down-regulated in N1 after drought treatment (Figure 2A, Supplementary Files S1 and S2).

When the DEGs of these comparison groups are represented by a Venn diagram, it was clear that both the unique and shared DEGs appeared between pairs (Figure 2B, Supplementary Files S1–S4). For example, 1611 DEGs (39.18% of the total) in d_N1 vs. CK_N1 were shared with the compared d_JD vs. CK_JD. In the comparison group d_JD vs. CK_JD, except for above 1611 DEGs, the remaining 4427 DEGs contain 1996 genes which have constitutive differences in the two soybean varieties under normal conditions. These results revealed that there were more genes involved in the response to drought stress in JD, among which there were many essential different genes that may be more relevant to the drought resistance soybean seedling.

### 2.4. Functional Classification of DEGs of Jindou 21 and Tianlong No.1 to Drought Stress

**Figure 3:**
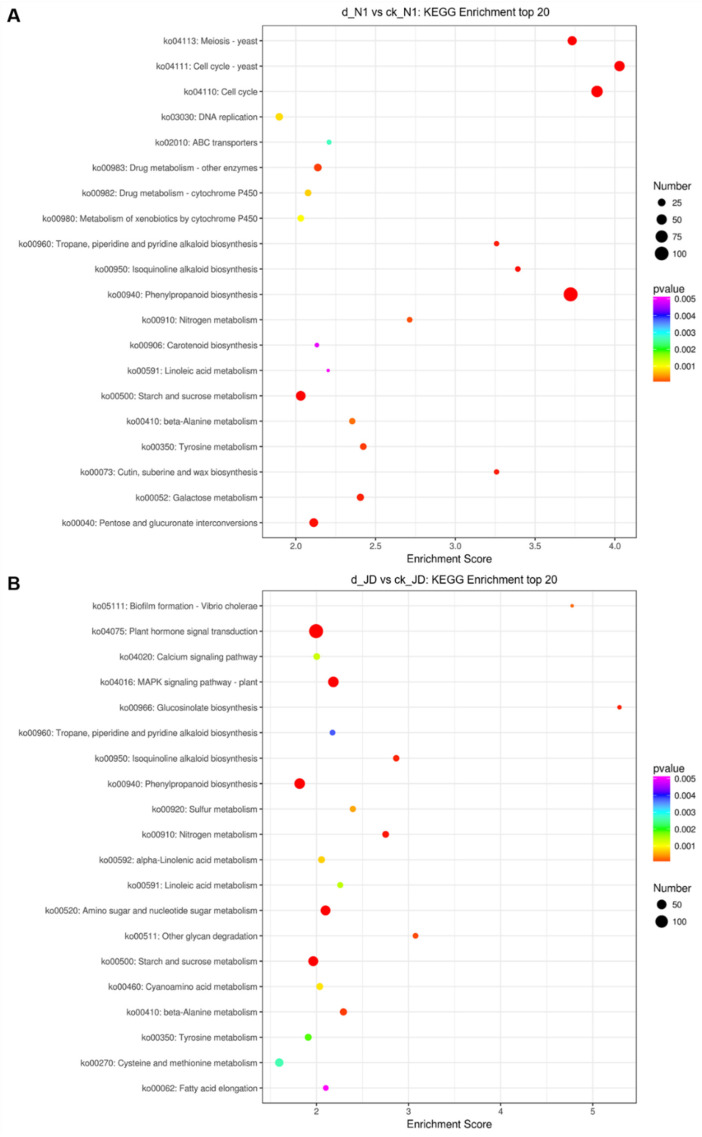
KEGG pathway analysis of DEGs of drought sensitive soybean N1 (**A**) and drought tolerance soybean JD (**B**) under the drought treatment, respectively. The *x* -axis indicates enrichment score, while the *y* -axis indicates pathway name. The size of the dot represents the number of DEGs. The different colors of dots represent different *p*-values.

In order to further explore the molecular response of JD and N1, we analyzed the function annotation of DEGs of JD and N1 after drought treatment (d_JD vs. CK_JD; d_N1 vs. CK_N1). The 2886 DEGs in N1 and 4069 DEGs in JD were assigned to the GO classification. They fall into three broad categories comprising biological process (BP), cellular component (CC), and molecular function (MF) (Table S3; Figure S2). The results showed that the DEGs of the JD and N1 were annotated to the almost same GO term, but many more DEGs in JD were annotated to each GO term. To understand which DEGs work to confer a specific pathway, KEGG pathway analysis was performed. We found that 1007 DEGs and 794 DEGs were enriched into 179 and 172 pathways in JD and N1, respectively (Table S3 and Supplementary Files S5 and S6). After drought stress treatment, many DEGs in JD were enriched into signal transduction pathways, including plant hormone signal transduction, calcium signaling pathway and MAPK signaling pathway-plant, while few DEGs in N1 were enriched into these pathways (Figure 3, Supplementary Files S7 and S8).

### 2.5. Validation of RNA-Seq Expression Levels by qRT-PCR

**Figure 4:**
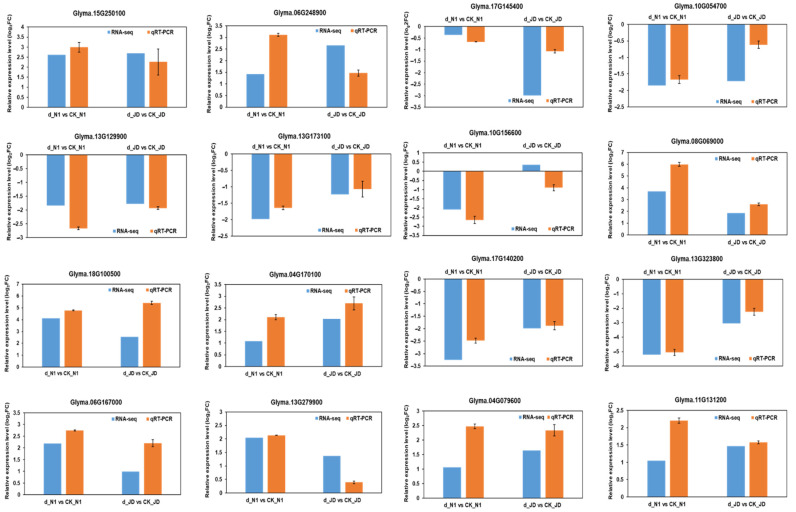
The comparison of selected genes’ expression pattern between RNA-seq and qRT-PCR under drought treatment. The *y* -axis indicates the log_2_FC values. FC represents the expression level fold change of genes in two soybean genotypes after drought treatment, respectively. Data represent the means ± SD of three biological replicates, and each biological replicate contained three technical replicates.

The total RNA of drought treatment and control of two soybean varieties JD and N1 provided a template for qRT-PCR verification. We randomly selected 16 DEGs to verify our RNA-Seq results. Figure 4 shows that the selected genes expression trend is almost consistent between qRT-PCR and RNA-Seq. The results showed a statistically significant positive correlation and coefficient of determination (R2) of 0.8725 was obtained between qRT-PCR and RNA-Seq (Figure S3). The above data indicated that the obtained RNA-Seq data are reliable.

### 2.6. Different Expression Patterns of Genes Related to Plant Hormone Signal Transduction under Drought Stress in Both Genotypes

**Figure 5:**
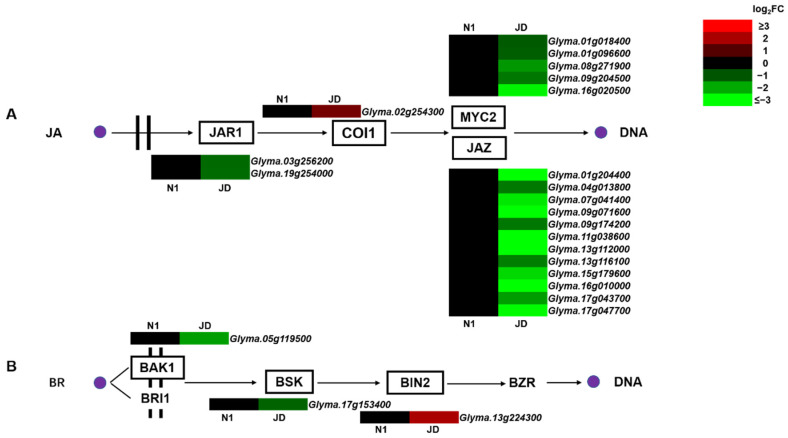
The phytohormone JA and BR signaling pathways and heatmap of related DEGs. (**A**,**B**) represent the signal transduction pathways of JA and BR and the heatmap of DEGs enriched in each pathway, respectively. The log_2_FC was colored using Microsoft Office Excel 2019 (red for up-regulated, green for down-regulated). The horizontal row represents a DEG with its gene ID, and the vertical columns represent d_N1 vs. CK_N1 and d_JD vs. CK_JD from left to right.

Previous studies have endorsed that endogenous plant hormones play an important role in regulating plant adaptation to drought conditions [10]. The present study found that JA and BR plant hormone signaling pathways were specially involved in the response to drought stress in drought-tolerant soybean variety JD (Figure 5A,B). Drought-tolerant JD has a large number of DEGs enriched in JA signaling pathway, and no DEGs in N1. For instance, 1 up-regulated *COI1*, 12 down-regulated *JAZ* genes and 5 down-regulated *MYC* genes were identified in JD after drought stress treatment (Figure 5A). Moreover, no DEGs of N1 enriched in BR signaling pathway and three DEGs (*BAKI1*, *BSK*, *BIN2*) in JD were identified (Figure 5B).

ABA, ET, AUXIN and SA signaling pathways were also involved in the response to drought stress in two soybean varieties (Figure S4). The several key components enriched in the above signaling pathways had significant differences between JD and N1. For example, there were three and seven up-regulated *ABFs* in ABA signal transduction in N1 and JD, respectively (Figure S4A). In ET and SA signaling pathways, one up-regulated *EIN3/EIL* and one down-regulated *NPR1* were enriched in drought-tolerant JD, but not in N1 (Figures S4B and S4D). The 3 and 11 differently expressed *AUXIN/IAA* genes were enriched in N1 and JD, respectively (Figure S4C).

### 2.7. Different Expression Patterns of Calcium Signaling Pathway and MAPK Signaling Pathway during Drought Stress in Both Genotypes

**Figure 6:**
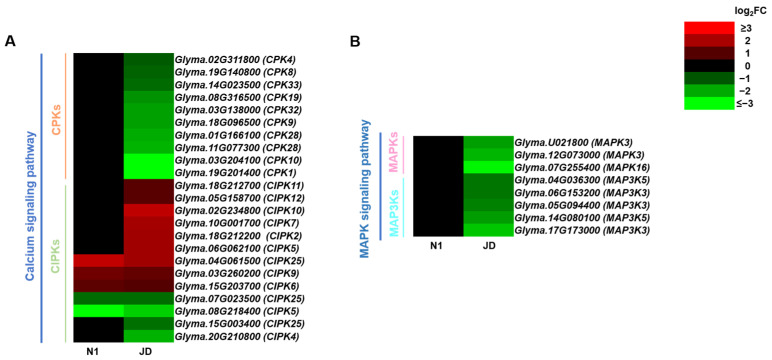
The heatmaps of differentially expressed genes enriched in the calcium signaling pathway (**A**) and MAPK signaling pathway (**B**). The log_2_FC was colored using Microsoft Office Excel 2019 (red for up-regulated, green for down-regulated). The horizontal row represents a DEG with its gene ID, and the vertical columns represent d_N1 vs. CK_N1 and d_JD vs. CK_JD from left to right.

In addition to plant hormones, calcium can be used as a second messenger to transmit drought stress signals to the cell membrane [30]. The current study showed that only 10 differentially expressed *CPKs* were down-regulated in drought-tolerant JD after drought treatment, while no differentially expressed *CPKs* were identified in drought-sensitive N1 (Figure 6A). In addition, 5 differentially expressed *CIPKs* (3 up-regulated and 2 down-regulated) were identified in drought-sensitive N1 and they were also identified in the JD (Figure 6A). Moreover, there are 8 *CIPKs* (6 up-regulated and 2 down-regulated) in drought-tolerant JD. The above results indicated that down-regulated *CPKs* and up-regulated *CIPKs* were induced by drought stress.

The mitogen-activated protein kinase (MAPK) cascade is one of the important conservative signal transduction pathways when plants convert extracellular drought stress signals into intracellular signals [31]. It is worth noting that *MAPK3s*, *MAPK16s*, *MAP3K3s* and *MAP3K5s* were significantly down-regulated in drought-tolerant variety JD after drought stress, whereas no DEGs related to MAPK cascade were identified in drought-sensitive N1 (Figure 6B). Hence, it is shown that MAPK signaling pathway is related to the drought tolerance of soybean seedling.

### 2.8. Different Expression Patterns of Transcription Factors during Drought Stress in Both Genotypes

**Figure 7:**
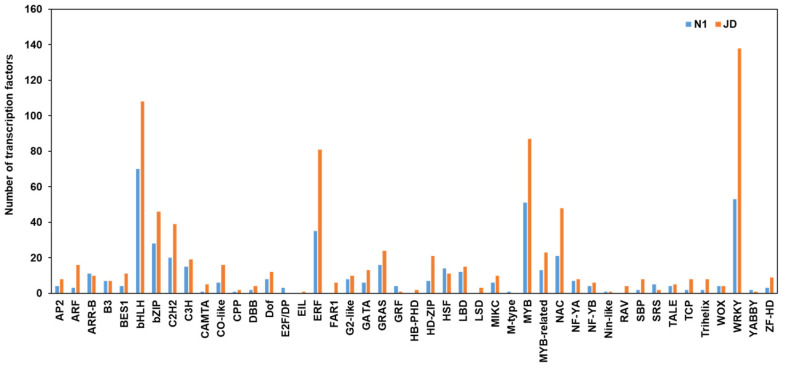
Classification and quantitative statistics of differentially expressed transcription factors (blue for N1; orange for JD). The *x* -axis indicates the categories of differentially expressed transcription factors. The *y* -axis indicates the number of differentially expressed transcription factors.

After the stress signal is transmitted into the cell through a series of signaling pathways, it could induce changes in the expression level of stress-related genes including TFs, which improves plant resistance to adverse environments [32]. To further reveal differences of drought tolerance between genotypes, we searched the different expressed TF genes after drought treatment in JD and N1, respectively. A total of 861 DEGs in JD were identified as TFs, among which 466 DEGs in N1 were identified (Supplementary Files S9 and S10). The differentially expressed TF genes encoding bHLH, WRKY, MYB, ERF, NAC were more than 55% of the total number of TFs in JD and N1 (Figure 7). We also found that the number of differentially expressed *WRKY*, *bHLH*, *MYB*, *ERF* in JD was bigger than that in N1 (Figure 7). Apart from that, other common TF families related to drought stress were also identified in two soybean varieties after drought stress, such as *NAC*, *MYB-related*, and *HD-ZIP*. These findings further indicated that a large number of TFs might participate in the process of plant drought tolerance, which could enhance the drought tolerance of soybean seedlings.

**Figure 8:**
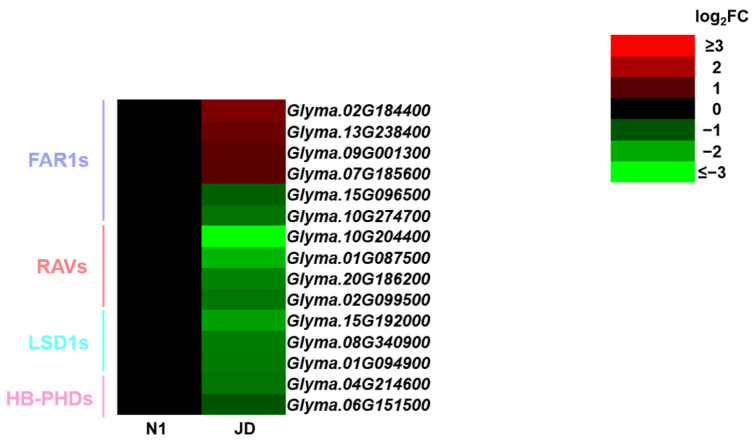
Heatmap of specific classes of differentially expressed transcription factors in drought-tolerant JD. The log_2_FC was colored using Microsoft Office Excel 2019 (red for up-regulated, green for down-regulated). The horizontal row represents a DEG with its gene ID, and the vertical columns represent d_N1 vs. CK_N1 and d_JD vs. CK_JD from left to right.

In addition, the present study found that five kinds of TFs are specifically expressed in JD under drought stress, including *FAR-RED IMPAIRED RESPONSE1 (FAR1)*, *Related to ABI3/VP1 (RAV)*, *Lesions Stimulating Disease1 (LSD1)*, *EIL*, and *HB-PHD*, which are only expressed in JD under drought stress. For example, six DEGs were identified as *FAR1s* in JD, including four up-regulated and two down-regulated genes (Figure 8). In this study, only four down-regulated *RAVs*, three down-regulated *LSD1* and two down-regulated *HB-PHDs* were identified in JD (Figure 8). Therefore, it is suggested that these specifically expressed TFs in drought-tolerant JD may play a crucial role in the drought tolerance at soybean seedling stage.

### 2.9. Drought Induces Differential Expression Profiles of Genes Related to Cell Wall Synthesis and Remodeling between JD and N1 Plants

**Figure 9:**
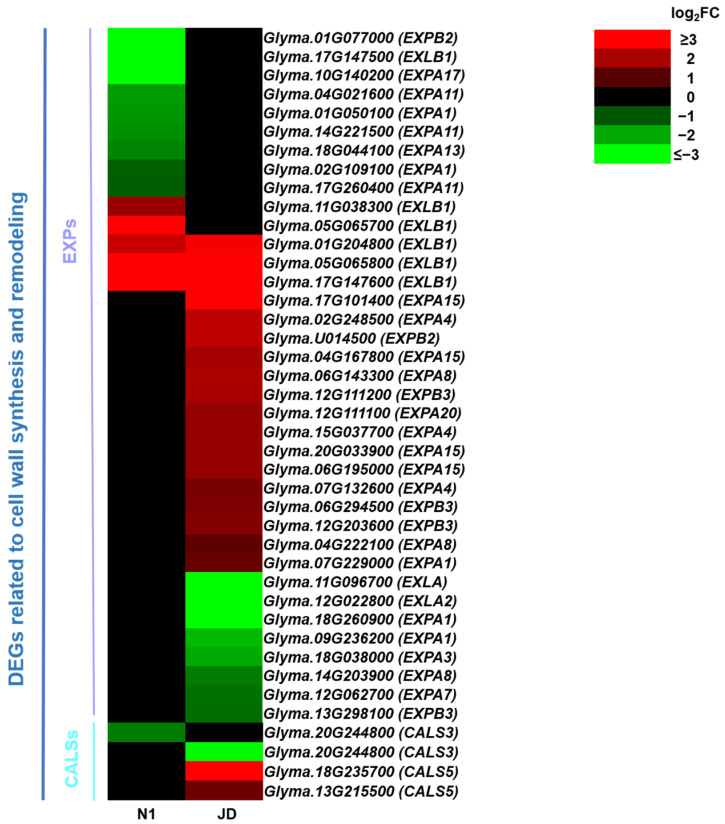
Heatmap of differentially expressed *EXPs* and *CALS* related to cell wall synthesis and remodeling. The log_2_FC was colored using Microsoft Office Excel 2019 (red for up-regulated, green for down-regulated). The horizontal row represents a DEG with its gene ID, and the vertical columns represent d_N1 vs. CK_N1 and d_JD vs. CK_JD from left to right.

In order to explore drought-tolerant genes related to cell wall synthesis and remodeling, we compared the differences of cell-wall-related DEGs between two soybean varieties. Subsequently, we analyzed the expression of *EXPs*, *CALSs PMEs* and *XTHs* in two soybean varieties under drought stress at seedling stage. Notably, 26 *EXPs* (18 up-regulated and 8 down-regulated) were identified in the drought-tolerant JD. There were 14 differentially expressed *EXPs* in drought-sensitive N1, of which 5 *EXPs* were up-regulated; 3 up-regulated *EXPs* were also up-regulated in JD, and the other 9 *EXPs* were down-regulated (Figure 9). In addition, further analysis showed that the expression of *CALS3* was down-regulated in both varieties, while the expression of *CALS5* was significantly up-regulated in JD (Figure 9). Moreover, our study showed that 17 and 21 *PMEs* were identified in drought-sensitive N1 and drought-tolerant JD under drought stress (Figure S5A). Through the analysis of the expression patterns of *XTHs* in two soybean genotypes with different drought tolerance, we found that the *XTHs* were significantly down-regulated by drought stress in soybean, and the number of down-regulated *XTHs* in JD was more than N1 (Figure S5B). The above results showed that a large number of up-regulated *EXPs* and *CALS5* in JD may produce a large amount of related proteins under drought stress, so that the cell wall could alleviate the damage caused by drought stress, and the drought tolerance of JD is stronger than N1 at seedling stage. Therefore, the up-regulation or down-regulation of genes related to cell wall synthesis and remodeling may enhance the drought tolerance of soybean seedlings through improving the physical structure of the cell wall, which made the drought tolerance of JD stronger than N1.

### 2.10. Analysis of DEGs Related to Stress Proteins in JD and N1 after Drought Treatment in Both Genotypes

**Figure 10:**
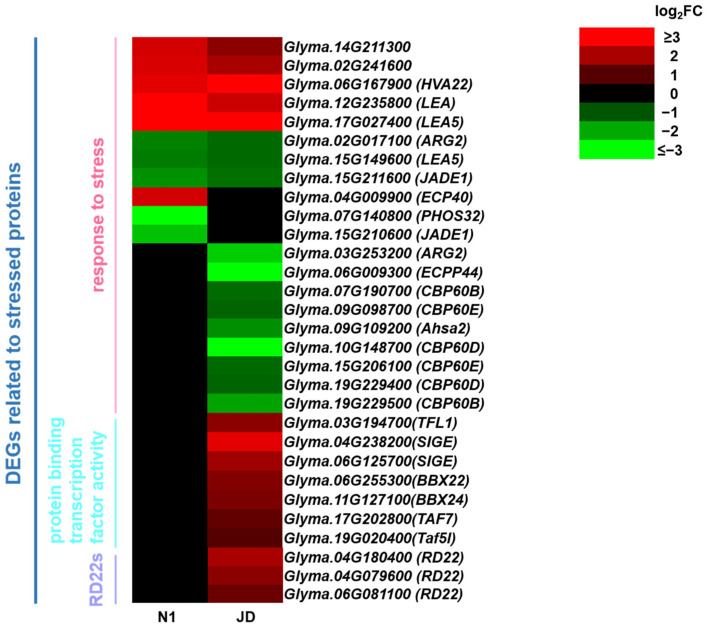
Heatmap of DEGs related to stressed proteins. The log_2_FC was colored using Microsoft Office Excel 2019 (red for up-regulated, green for down-regulated). The horizontal row represents a DEG with its gene ID, and the vertical columns represent d_N1 vs. CK_N1 and d_JD vs. CK_JD from left to right.

When the drought stress signal is transmitted to the nucleus, stress-related TFs are activated, which regulates the expression of stress-related protein genes. To reveal the functional genes of soybean seedling tolerance to drought stress, we analyzed the DEGs enriched in GO term “response to stress” (GO:0006950) in two soybean varieties after drought treatment. The results showed that 17 DEGs in JD and 11 DEGs in N1 were enriched into GO term “response to stress” (GO:0006950), and among them 8 DEGs were enriched in both JD and N1. It was further explained that only three specific DEGs (*Glyma.04G009900*, *ECP40*; *Glyma.07G140800*, *PHOS32*; *Glyma.15G210600*, *JADE1*) in N1 were enriched into the term, while nine specific DEGs (*Glyma.03G253200*, *ARG2*; *Glyma.06G009300*, *ECPP44*; *Glyma.07G190700*, *CBP60B*; *Glyma.09G098700*, *CBP60E*; *Glyma.09G109200*, *Ahsa2*; *Glyma.10G148700*, *CBP60D*; *Glyma.15G206100*, *CBP60E*; *Glyma.19G229400*, *CBP60D*; *Glyma.19G229500*, *CBP60B*) (Figure 10) in JD were enriched in this term. Meanwhile, we also found that the GO term “protein binding transcription factor activity”, enriched seven DEGs (*Glyma.03G194700*, *TFL1*; *Glyma.04G238200*, *SIGE*; *Glyma.06G125700*, *SIGE*; *Glyma.06G255300*, *BBX22*; *Glyma.11G127100*, *BBX24*; *Glyma.17G202800*, *TAF7*; *Glyma.19G020400*, *TAF5L*) and was only induced by drought stress in drought-tolerant JD (Figure 10). In addition, we identified three up-regulated DEGs, *RD22s*, *Glyma.04G180400*, *Glyma.04G079600 and Glyma.06G081100*, in drought-tolerant JD (Figure 10). Interestingly, no differentially expressed *RD22* was identified in drought-sensitive N1. Therefore, it is speculated that there is a significant difference in calmodulin-binding proteins (*CBPs)*, protein-binding transcription factor activity (*SIGEs*, *BBXs, TAFs*) and *RD22s* in drought-tolerant JD and N1 under drought treatment, which gives a stronger drought tolerance to JD than that in N1 at seedling stage.

## 3. Discussion

In this research, two soybean cultivars with different drought tolerance, JD and N1, were selected, and we inferred that the drought tolerance of JD was significantly stronger than that of N1 based on the identification results of the drought tolerance phenotype and the survival rate at the seedling stage (Figure 1). Meanwhile, transcriptome sequencing technology was used to compare and analyze the mechanisms of these two soybean cultivars in response to drought stress at the seedling stage. A total of 6038 and 4112 DEGs were identified in drought-tolerant JD and drought-sensitive N1, respectively. Subsequent analysis using KEGG and GO analysis identified key drought stress signal transduction pathways and potential genes. The results showed that more signaling pathways were involved in the response to drought stress in JD, including BR, JA, calcium and MAPK signaling pathways. In addition, more DEGs encoding TFs, cell wall metabolism and stress-related proteins were found to be involved in the drought tolerance of soybean seedlings.

### 3.1. Plant Hormone Signal Transduction Plays a Vital Role in Drought Tolerance of Soybean Seedlings, Especially JA and BR

Drought stress changes the endogenous biosynthesis and signal transduction of various plant hormones [8]. Many studies have reported that the expression of *ABF* in ABA signaling pathway was induced by drought stress and positively regulated the drought tolerance of plants [33]. In agreement with these studies, our study identified that *ABFs* were up-regulated by drought treatment in both JD and N1, but the number of up-regulated *ABFs* in JD was much more than N1. Several studies have shown that *EIN3/EIL* in ET signaling pathway could promote the drought tolerance in plants [34]. Similarly, our study found that an *EIN3/EIL* gene in JD was up-regulated four times when the JD was challenged by drought stress, while there were no changes of *EIN3/EIL* gene in N1. The TIR1/AFB-Aux/IAA-ARF signaling system is the main pathway of plant auxin signal transduction [35]. The Aux/IAA proteins make a valuable contribution towards auxin signaling as auxin co-receptors and transcriptional repressors. In *Arabidopsis*, auxin-sensitive Aux/IAA proteins mediate drought tolerance by regulating the levels of glucosinolates [13]. OsIAA20, an Aux/IAA protein, may be a positive regulator for drought tolerance involved in ABA signaling pathways [36]. Different from previous studies, our results identified six up-regulated and seven down-regulated *AUX/IAA* genes in JD, and only three up-regulated genes in N1. These results determined that *Aux/IAA* in JD may be involved in the regulation of the soybean drought tolerance in a more complex mode. ABA, ET and AUXIN hormones are endogenous hormones related to plant drought tolerance.

Interestingly, our research found that JA and BR signaling pathways are likely to be involved in the response of soybean seedlings to drought stress in drought-tolerant variety JD. This result has not been reported in previous studies on the mechanism of drought tolerance of soybean seedlings. JA is catalyzed by JAR1 and converted to biologically highly active specific enantiomer of JA-Ile [37]. COI1 acts as a JA-Ile receptor in JA signal pathway [38]. JAZ proteins are regarded as the switch of JA signaling pathway and play a key role in JA signal pathway. The previous studies have determined that overexpression of *OsJAZ1* can attenuate the drought tolerance of rice through regulating JA and ABA signal pathway [39]. At present, there are few studies on the functional identification of plant MYCs in drought tolerance, especially in soybean. The present study indicated that soybean seedling response to drought stress resulted in the 1 up-regulated *COI1*, which led to 12 down-regulated *JAZs*, as well as the 5 down-regulated *MYC* in drought-tolerant JD. The plant steroid hormone BR not only plays a role in plant growth and development, but also participates in plant tolerance to stress treatments, including drought stress [40]. BR binds to the membrane-localized leucine-rich repeat receptor kinase BRI1 on the cell membrane and then binds to the coreceptor BAK1 to transmit the BR signal through BSK, BIN2, and BZR [41,42,43,44]. Previous research has endorsed that BIN2 could potentiate the drought tolerance of *Arabidopsis* by phosphorylating RD26 [14]. Consistent with this observation, our study also revealed the *BIN2*, which is enriched in BR signal pathway in JD, is up-regulated and may further activate the expression of other downstream genes to enhance the soybean drought tolerance. As for the research on the functions of *BAKI1* and *BSK* in soybean seedlings in response to drought stress, further research is needed on the relationship between the down-regulated expression patterns of these genes and the drought tolerance of soybean seedlings. The above results demonstrated that the difference of drought tolerance between JD and N1 may be due to the JA and BR plant hormone involved in stress regulation. Taken collectively, we inferred that these DEGs enriched in JA and BR signal pathway may play a crucial role in the drought tolerance of soybean seedling.

### 3.2. Protein Kinases Related to Calcium and MAPK Signaling Pathway under Drought Conditions

When challenged by drought stress, plant cells can sense stress signals and transmit them from extracellular to intracellular through different transduction pathways, in which calcium and MAPK signal cascades are two important transduction pathways. Calcium (Ca^2+^) is the important second messenger in plant cells, and its signals are involved in the regulation of plant growth and development, as well as tolerance to drought, salt, low temperature stress, pathogens and other processes [45]. In plants, it has been identified that the specific calcium sensor-protein kinase effector module is encoded by either the calcium-dependent protein kinases (CPKs or CDPKs) or CBL-interacting protein kinase (CIPKs) kinase effector [46]. Many studies have found that *CPKs* play a positive role in regulating abiotic stress tolerance such as drought and salt stress in *Arabidopsis*, maize, and rice [47,48,49]. The current study identified 10 down-regulated *CPKs*. The relationship between *CPK* genes and stress response in soybean may be different from that in *Arabidopsis* and rice, resulting in different response patterns. Xu et al., have suggested that *GmCIPK2* positively regulates drought tolerance and ABA signal transduction in soybean [50]. Consistent with the above study, this study found that there were more up-regulated *CIPK* genes in JD than in N1. Hence, our study inferred that the drought response patterns and functions of *CPKs* and *CIPKs* may be more complicated in soybean seedlings than other plants.

MAPK cascades are activated when stress signals are transmitted to the intracellular [51]. MAPK cascades are minimally composed of three crucial kinases: MAPKs, MAPK kinases (MAPKKs, MAP2Ks or MEKs) and MAPK kinase kinases (MAPKKKs or MAP3Ks) [52]. In tomato, *SlMPK4* positively regulates tomato tolerance to drought stress [53]. The cotton *GhMPK6a* negatively monitors osmotic tolerance and bacterial infection in transgenic tobacco [54]. Consistent with these studies, it was identified that three down-regulated *MPKs* genes were differentially expressed only in JD, while there were no *MPKs* in N1. Many studies have shown that the function of *MAP3Ks* in plant response to drought stress is also inconsistent. For instance, the overexpression of *AtMAP3K18* significantly enhanced the drought tolerance of *Arabidopsis* [55]. In cotton, *GhMAP3K40* negatively controls plant growth and abiotic stress tolerance [56], while *GhMAP3K14* positively regulates the drought tolerance of cotton [57]. The study only identified five down-regulated *MAP3Ks* genes in the drought-tolerant JD and none in N1. Hence, it can be suggested that the significantly down-regulated *MAPKs* and *MAP3Ks* genes in MAPK signaling pathway may be due to the drought tolerance of soybean seedling and one of reasons for the difference in drought tolerance of JD and N1.

### 3.3. Transcription Factor (TF) Related Genes Are a Critical Component of Drought Response Machinery at Soybean Seedling Stage

The drought stress response is controlled by complex regulatory networks in plants. Transcription factors (TFs) are important regulators in this network, which play a pivotal role by activating or inhibiting the expression of downstream stress-related target genes [15]. Up to now, it has been reported that many TFs are involved in the regulation of drought stress tolerance in soybean. TFs GmWRKY27 interacts with GmMYB174 and jointly inhibits the expression of downstream target gene GmNAC29, which enhances the drought tolerance of soybean [58]. Yang et al., have shown that GmNAC9 is a positive regulator of drought stress response and can improve the drought tolerance in soybean [59]. Previous studies have revealed that several kinds of TFs, including MYB, WRKY, HD-ZIP, bZIP, bHLH, and DREB, are involved in soybean drought tolerance using transcriptome analysis [25,26,27]. Similarly, our studies have identified these common drought-related TFs. In addition, we also identified that five kinds of TFs were induced in drought-tolerant JD but not in N1, including *EIL*, *FAR1*, *RAV*, *LSD1*, and *HB-PHD*. The transcription factor *EIL* changes as described in the above ET signal pathway. It has been proved that *AtFAR1* can respond strongly to drought stress while *FAR1* can positively regulate the ABA signal pathway and integrate light signal and ABA signal together, in order to give plants better adaption to environmental stress [60]. *RAV* is a multifunctional regulator to plant growth, leaf senescence and response to abiotic stresses such as drought and salt stress [61]. Previous reports have shown that *LSD1* is associated with soybean defense against root parasites [62]. These studies indicated that those specific TFs may play essential roles in plant response to environmental stress. Therefore, the different expression of these specific TFs only in drought-tolerant variety JD may be one of the pivotal reasons why JD is more drought tolerant than N1.

### 3.4. Cell Wall Metabolism and Stress-Related Proteins Are Vital for Soybean Seedling Survival under Drought Conditions

In order to survive and protect themselves against water deficit conditions, the plant cell wall must be remodeled rapidly to maintain flexibility; EXPs are involved in this process [63]. Several studies have shown that *EXPs* can positively regulate the abiotic tolerance of plants, including wheat and soybean [64,65]. Similarly, our study revealed that there were 18 up-regulated *EXPs* in drought-tolerant JD, and only 5 up-regulated *EXPs* in drought-sensitive N1. In addition, there were some down-regulated *EXP* genes in two varieties. The reason for this may be that different *EXP* genes respond to drought stress sooner or later. Meanwhile, it is generally believed that callose is produced by callose synthase (CALS) in the cell walls of many higher plants and plays an important role in plant development and/or response to a variety of biotic and abiotic stress [66,67] The present study showed that the expression of one *CALS3* was down-regulated in both varieties, while the expressions of two *CALS5* were significantly up-regulated in JD. Therefore, we speculated that a large number of differentially expressed *EXPs* and *CALSs* may regulate the cell wall synthesis and remodeling of drought-tolerant JD under drought stress to reduce the damage of cell wall, and thus JD had stronger drought tolerance.

When subjected to drought stress, plants can produce stress-related proteins to resist the harm caused by drought stress. In this study, we analyzed the DEGs enriched in “response to stress” (GO:00006950) and found that there were nine specific DEGs including six *CBPs* in JD. AtCBP is a negative regulator of osmotic stress tolerance in seedlings [68]. Therefore, we hypothesized that down-regulated *CBPs* in JD may be related to the increased drought tolerance of JD. In addition, a previous study has shown that stress-related protein gene *GmRD22* can be induced by salt, ABA and PEG, and can improve the stress tolerance of plants [69]. In this regard, our study only found three up-regulated *RD22* in the drought-tolerant JD, while no *RD22* in the drought-sensitive N1. Therefore, we speculated that the significantly up-regulated *RD22* makes drought-tolerant JD accumulate more RD22 protein than drought-sensitive N1, which might be a key factor contributing to the drought tolerance of soybean seedlings.

### 3.5. A Schematic Model of Soybean Seedling Response to Drought Stress in Two Cultivated Soybeans

**Figure 11:**
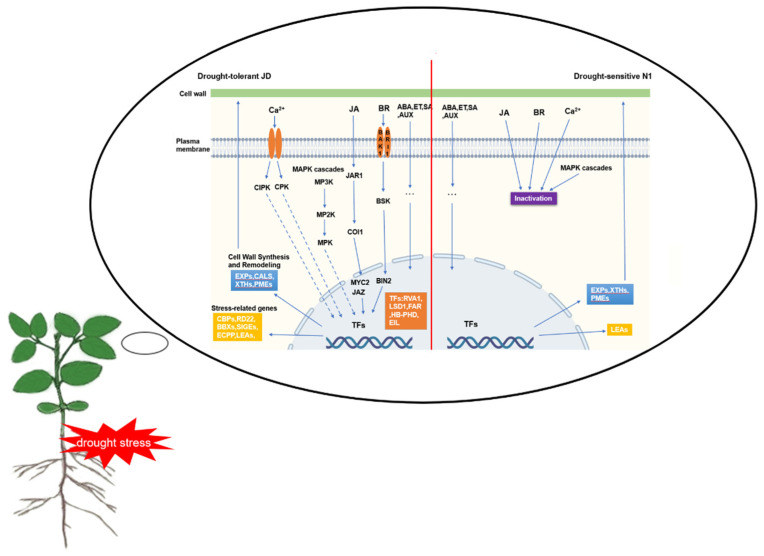
A schematic model of drought-tolerant and drought-sensitive soybean seedling genotypes under drought stress. This model was described based on crucial putative components of drought response identified in this study, supported by previously described schemes of plant abiotic stress response pathways/networks. The left and right parts of red line represent the responsive drought stress in drought-tolerant soybean Jindou 21 and drought-sensitive soybean Tianlong No.1, respectively. Key to abbreviations: JA—Jasmonic acid, BR- Brassinosteroids, ABA—Abscisic acid, ET—Ethylene, SA—Salicylic acid, AUX—Auxin, JAR1- Jasmonate resistant 1, COI1—Coronatine insensitive 1, JAZ—Jasmonate-ZIM domain, BRI1—Brassinosteroid insensitive, BAK1—BRI1 Associated kinase1, BSK—BR signaling, BIN2—Brassinosteroid insensitive2, MPK—Mitogen-activated protein kinase, MP2K—MAPK kinase, MP3K—MAPK kinase kinases, CDPKs—Calcium dependent protein kinase, CIPKs—CBL-interacting protein kinase, RAV1—Related to ABI3/VP1, LSD1—Lesions Stimulating Disease1, FAR1—Far-Red Impaired Response1, EIL—Ethylene insensitive 3-like, HB-PHD- Homeobox gene, XTHs—Xyloglucan endotransglucosylase/hydrolases, EXPs—Expansins, CALS- Callose synthase, PME—Pectin methylesterase, CBPs—Calcium binding proteins, RD22s—Dehydration responsive proteins, BBXs—B-box zinc finger proteins, ECPPs—Embryogenic cell phosphoproteins, SIGEs- Sigma factor genes, LEAs- Late embryogenesis abundant proteins.

Based on the main findings of the drought-responsive DEGs and their associated pathways/networks, we proposed a schematic model presented in Figure 11. This model shows a hypothetical picture revealing the cell wall modifying enzymes, plant hormones, Ca^2+^, TF activities, and stressed proteins, as well as signaling terms which increased the drought tolerance of soybean seedlings compared with the drought-sensitive soybean genotype.

## 4. Materials and Methods

### 4.1. Plant Materials, Growth Conditions and Drought Stress

Two soybean cultivars, Jindou 21 and Tianlong No.1, with different drought tolerance levels were selected for the research. Seeds were germinated on vermiculite in 10 cm × 10 cm square pots in a greenhouse. The environmental settings for soybean growth were 25 °C with a photoperiod of 16 h/8 h (light/dark). Four 2-week-old seedlings (V2–V3 stage) in each pot were used for drought treatment. For each pot, the vermiculite was fully watered before drought treatment [59]. The last watering date before drought stress was recorded as control (0 d). The root, stem and leaf tissue were respectively taken as control samples on 0 d and drought samples on 6 d of drought treatment, which were placed rapidly into liquid nitrogen and then stored at −80 °C for RNA extraction. Phenotypic photographs were taken at 0 d, 7 d, 8 d and 9 d during drought treatment, and the survival rate was counted at rewater on 9 d of drought treatment.

### 4.2. Total RNA Extraction, Library Construction, Sequencing and Mapping

For each RNA extraction, frozen tissues from one individual plant were homogenized and up to 100 mg was used. Total RNA was extracted using the mirVana miRNA Isolation Kit (Ambion, Austin, Tex, USA) following the manufacturer’s protocol. Equal quantities of RNA from roots, stems and leaves samples at 0 d and 6 d were pooled to construct a complementary DNA (cDNA) library. RNA integrity was evaluated using the Agilent 2100 Bioanalyzer (Agilent Technologies, Santa Clara, CA, USA). The samples with RNA Integrity Number (RIN) ≥ 7 were subjected to the subsequent analysis. The libraries were constructed by TruSeq Stranded mRNA LTSample Prep Kit (Illumina, San Diego, CA, USA) according to the manufacturer’s instructions. Then, these libraries were sequenced on the Illumina sequencing platform (Illumina HiSeq X Ten) and 125 bp/150 bp paired-end reads were generated. Raw data (raw reads) were processed using Trimmomatic [70]. The reads containing ploy-N and the low-quality reads were removed to obtain the clean reads. Meanwhile, the guanine-cytosine (GC) content and quality scores (Q20, Q30) of the clean data were calculated. Then, the clean reads were mapped to reference genome Gmax_275_v2.0 (https://genome.jgi.doe.gov/portal/pages/dynamicOrganismDownload.jsf?organism=Phytozome (accessed on 1 March 2019)) location information; the reference genome or gene, as well as the unique sequence characteristic information of the sequencing sample, were obtained through hisat [71]. The library construction and sequencing were carried out at OE Biotech (Shanghai, China). The total of RNA-seq raw data has been submitted to the NCBI BioProject with the SRA accession number PRJNA813355.

### 4.3. Transcriptomic Data Analysis

In transcriptome sequencing analysis, the gene expression level is estimated by counting the sequencing reads located in the genomic region or gene exon region. Using the known reference gene sequences and annotation files as the database, we used the method of sequence similarity alignment to identify the expression abundance of each gene in each sample. Gene expression levels were calculated using the “Fragments Per kb Per Million Reads” (FPKM) method that is the number of fragments per thousand bases from a gene per million fragments [72]. FPKM value of each gene was calculated using cufflinks [73], and the read counts of each gene were obtained by htseq-count [74]. Principal component analysis (PCA analysis) of gene expression was used to investigate the distribution of samples, and explore the relationship between samples or verify the experimental design [75]. DESeq software was used to standardize the counts number of each sample gene (baseean value was used to estimate the expression) and the fold of difference [76]. Meanwhile, NB (negative binomial distribution test) was used to test the significance of reads number. In the study, DEGs were identified using the DESeq [77] R package functions estimated SizeFactors and nbinomTest and analyzed as described previously. In addition, the *p* value < 0.05 and an absolute value of |log_2_FoldChange| > 1 were selected as the thresholds to judge the significance of differences in gene expression.

### 4.4. Functional Classification of DEGs

Gene Ontology (GO) knowledgebase is the largest gene function information source in the world, including three GO term categories: molecular function (MF), cellular component (CC), and biological process (BP). Kyoto Encyclopedia of Genes and Genomes (KEGG) is a database resource that can understand the advanced functions and utility of biological systems from the large-scale molecular data generated by genome sequencing. GO enrichment and KEGG pathway enrichment analysis of DEGs were respectively performed using R based on the hypergeometric distribution [78].

### 4.5. Transcription Factor Analysis

Plant TFDB 4.0 contains TFs identified from 165 species, providing a comprehensive genomic TF spectrum of green plant. Based on the TF sequence derived from Peking University transcription factor database (http://planttfdb.cbi.pku.edu.cn/ (accessed on 15 September 2019)), we analyzed and classified all the DEGs after drought treatment in JD and N1, separately [79].

### 4.6. Quantitative Real-Time PCR (qRT–PCR) Verification

Gene-specific primers for qRT-PCR were designed in the GenScript Real-time PCR Primer Design (https://www.genscript.com/tools/real-time-pcr-taqman-primer-design-tool (accessed on 2 April 2021)) and are listed in Table S4. Template cDNAs were synthesized using the Prime Script™ RT Reagent Kit (TaKaRa, Kyoto, Japan) from 1.0 µg of total RNAs following the manufacturer’s instructions. ChamQ Universal SYBR qPCR Master Mix (Q711, Vazyme, Nanjing, China) was used as the labeling agent, while the soybean actin gene *GmActinⅡ (Glyma.18g290800)* served as the internal reference gene. These reactions were conducted by using a Light Cycler 480 instrument. Sample cycle threshold (CT) values were standardized for each template based on reference gene control primer reaction, and the 2^–ΔΔCT^ method was used to analyze relative changes of gene expression [80]. Three independent biological replicates were performed for each sample to ensure statistical credibility.

## 5. Conclusions

In this study, the drought tolerance of two cultivated soybean varieties was identified. RNA-seq technique was used to analyze and compare the DEGs of the two soybean varieties before and after drought treatment, which explained the difference in drought tolerance between the two varieties by identifying related drought stress signal transduction pathways and key genes. Our study showed that the difference in drought tolerance between the drought-tolerant cultivar JD and the drought-sensitive cultivar N1 may be due to the fact that JD could transmit drought stress signals to the cell and nucleus through various signal transduction pathways (including JA, BR, Calcium and MAPK). In this process, numerous stress-related TFs are activated which can bind to the promoter region of the downstream target genes, then activate or inhibit the expression of downstream stress-related genes (such as *EXPs*, *CBPs*, *BBXs* and *RD22s*). These stress-related proteins are processed by the endoplasmic reticulum and transported to specific regions to perform their functions. These above DEGs will provide candidate gene resources for the study of drought tolerance in the soybean seedling stage.

## Supplementary Materials

The following supporting information can be downloaded at: https://www.mdpi.com/article/10.3390/ijms23052893/s1.
