# Supplementary material for: Key Soybean Seedlings Drought-Responsive Genes and Pathways Revealed by Comparative Transcriptome Analyses of Two Cultivars
Source: Int J Mol Sci. 2022 Mar 7;23(5):2893. doi: 10.3390/ijms23052893 (PMC8911164; doi:10.3390/ijms23052893)
Supplement: Supplementary file 1 [file ijms-23-02893-s001.zip › Supplemental Materials 20220211/Supplemental figure and table.pptx]

## Slide 1
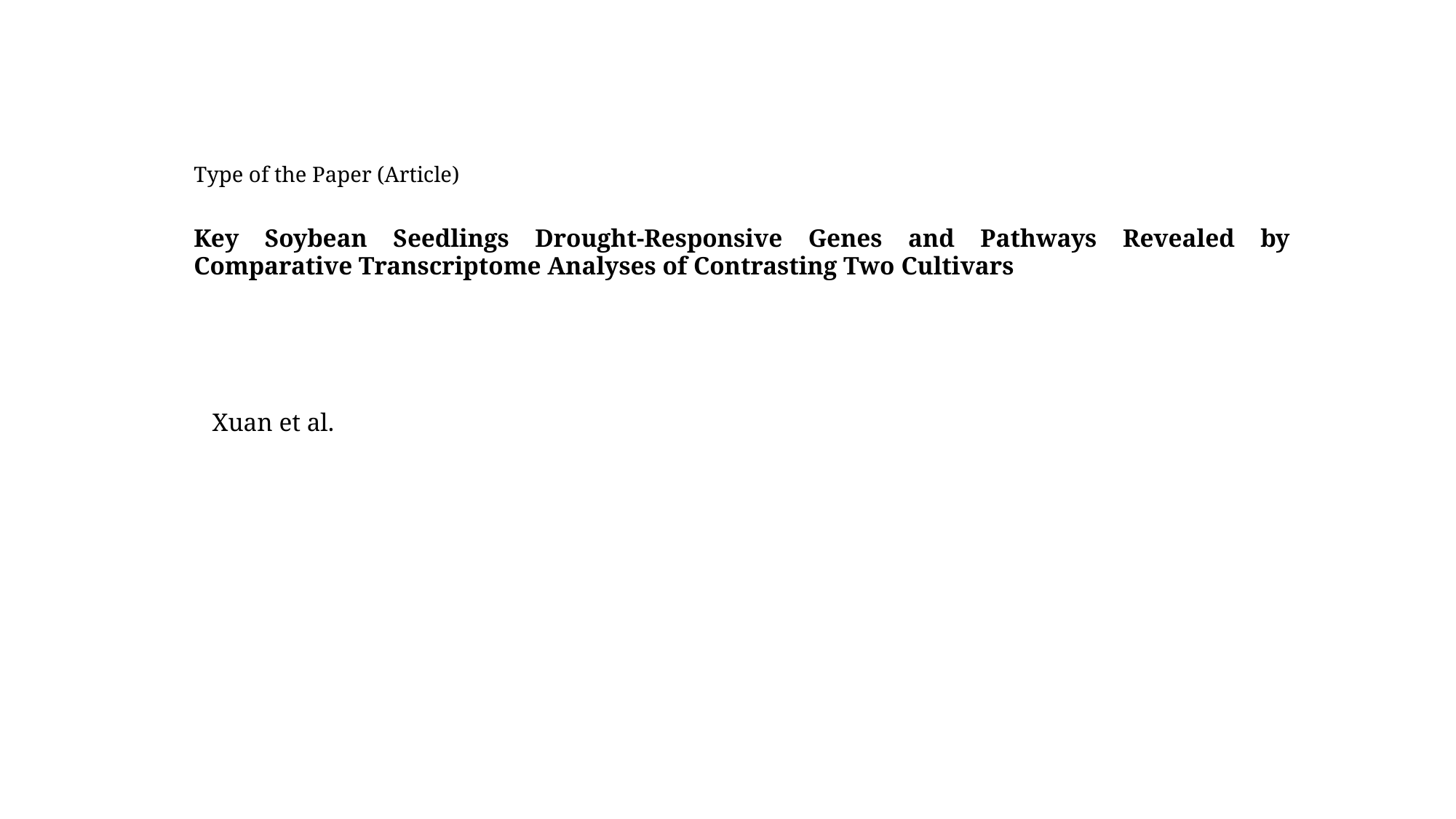

Type of the Paper (Article)
Key Soybean Seedlings Drought-Responsive Genes and Pathways Revealed by Comparative Transcriptome Analyses of Contrasting Two Cultivars
Xuan et al.

## Slide 2
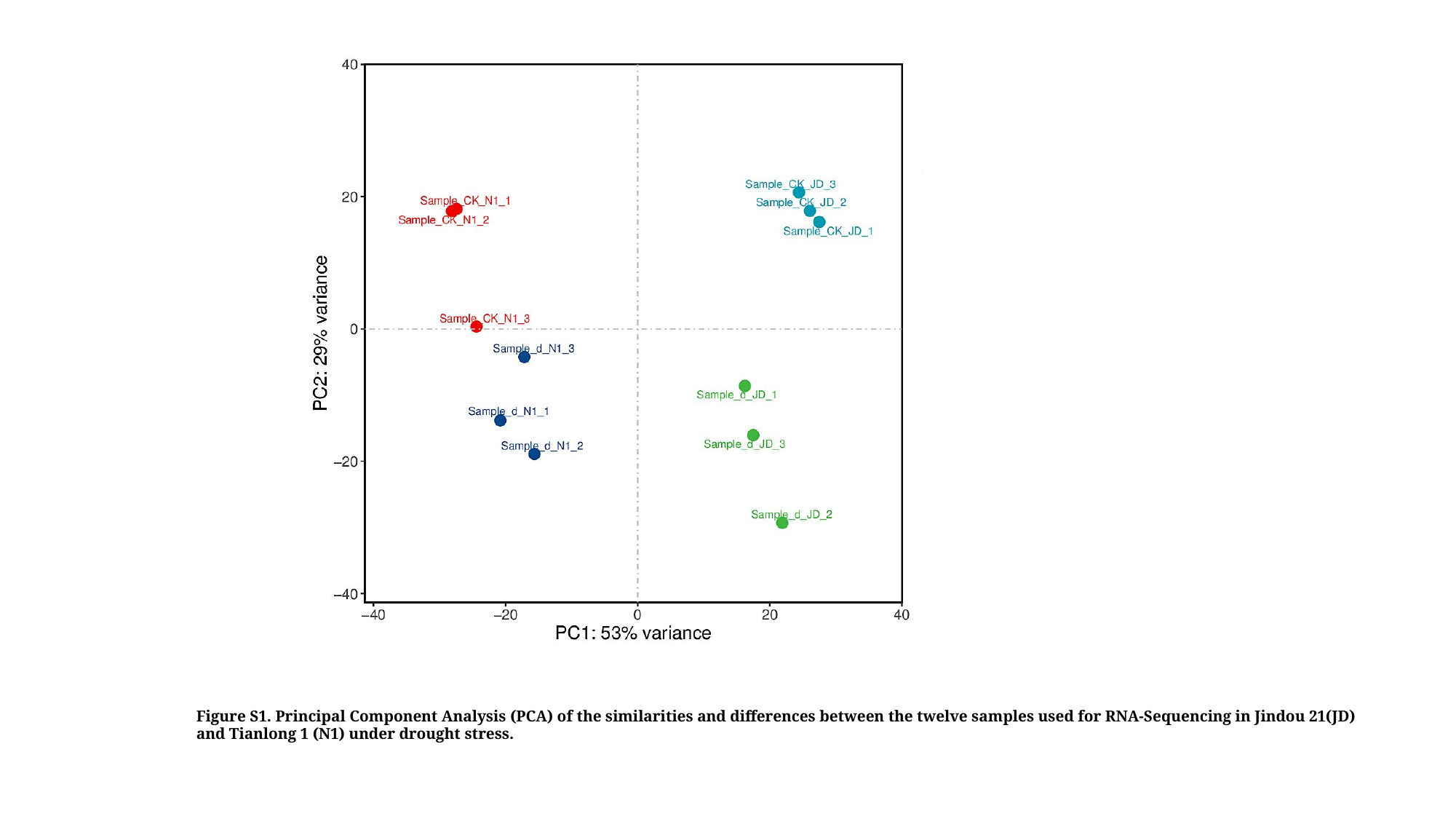

Figure S1. Principal Component Analysis (PCA) of the similarities and differences between the twelve samples used for RNA-Sequencing in Jindou 21(JD) and Tianlong 1 (N1) under drought stress.

## Slide 3
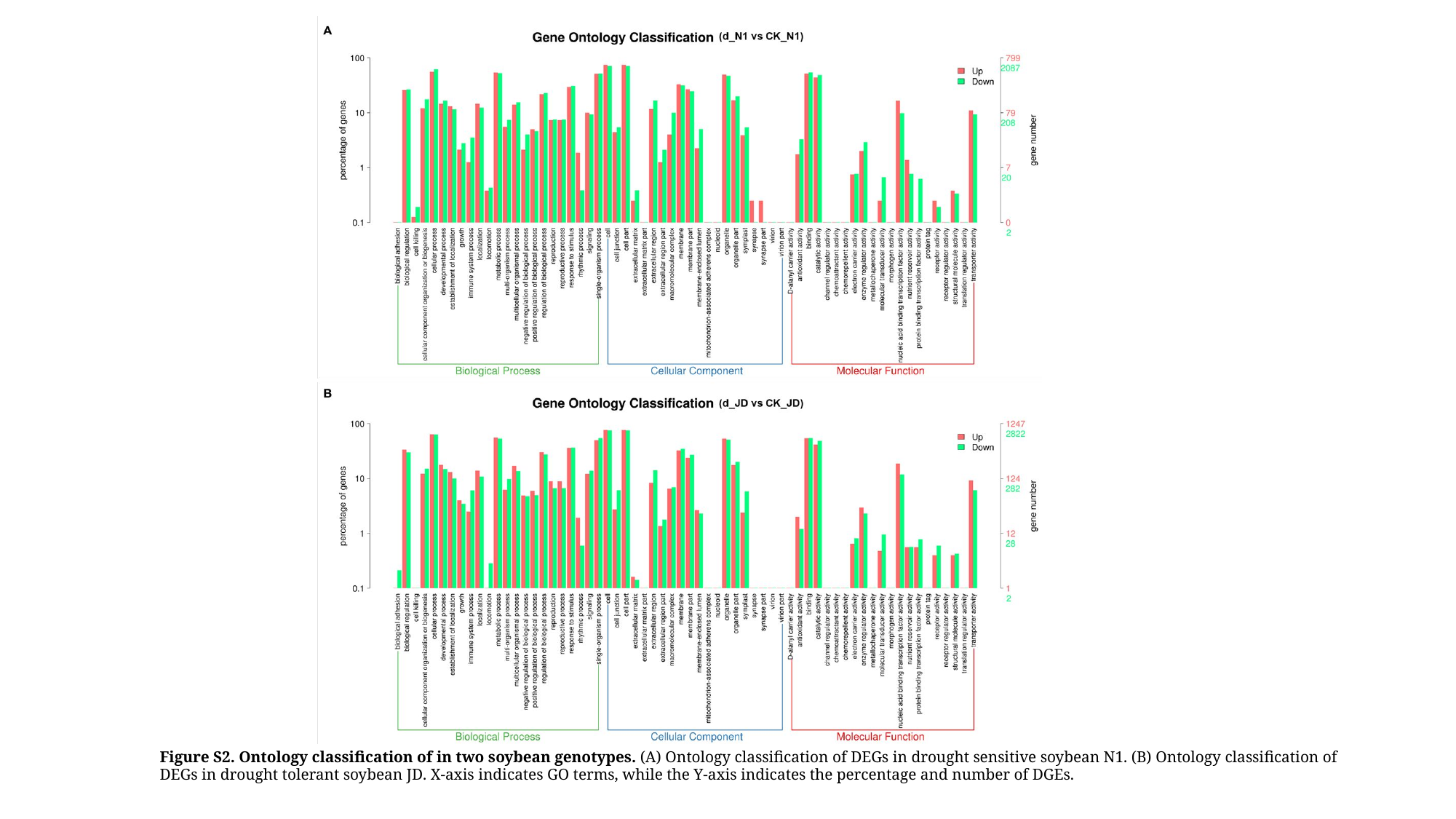

Figure S2. Ontology classification of in two soybean genotypes. (A) Ontology classification of DEGs in drought sensitive soybean N1. (B) Ontology classification of DEGs in drought tolerant soybean JD. X-axis indicates GO terms, while the Y-axis indicates the percentage and number of DGEs.

## Slide 4
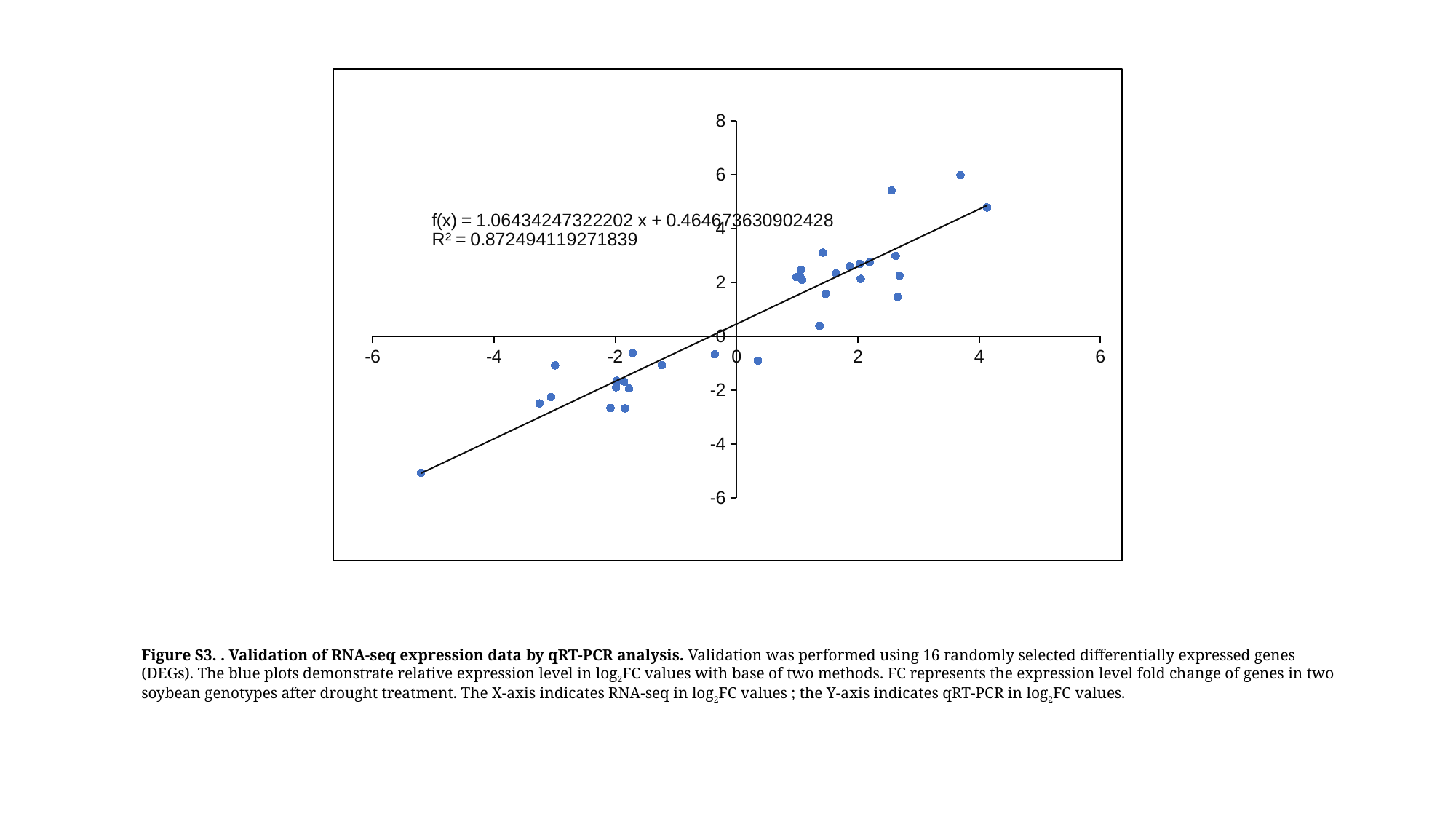

### Chart
| Category | |
|---|---|Figure S3. . Validation of RNA-seq expression data by qRT-PCR analysis. Validation was performed using 16 randomly selected differentially expressed genes (DEGs). The blue plots demonstrate relative expression level in log2FC values with base of two methods. FC represents the expression level fold change of genes in two soybean genotypes after drought treatment. The X-axis indicates RNA-seq in log2FC values ; the Y-axis indicates qRT-PCR in log2FC values.

## Slide 5
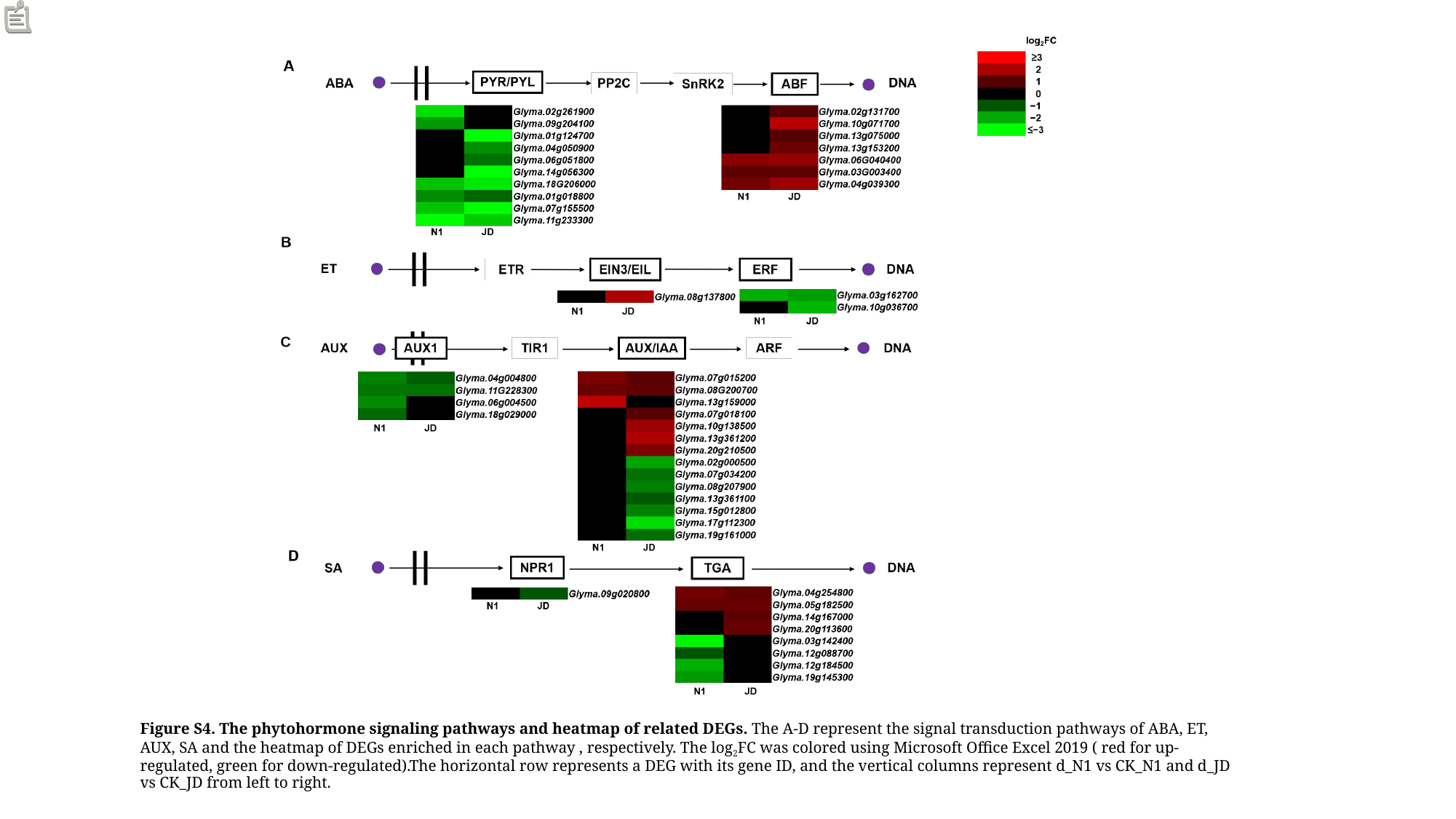

Figure S4. The phytohormone signaling pathways and heatmap of related DEGs. The A-D represent the signal transduction pathways of ABA, ET, AUX, SA and the heatmap of DEGs enriched in each pathway , respectively. The log2FC was colored using Microsoft Office Excel 2019 ( red for up-regulated, green for down-regulated).The horizontal row represents a DEG with its gene ID, and the vertical columns represent d_N1 vs CK_N1 and d_JD vs CK_JD from left to right.

## Slide 6
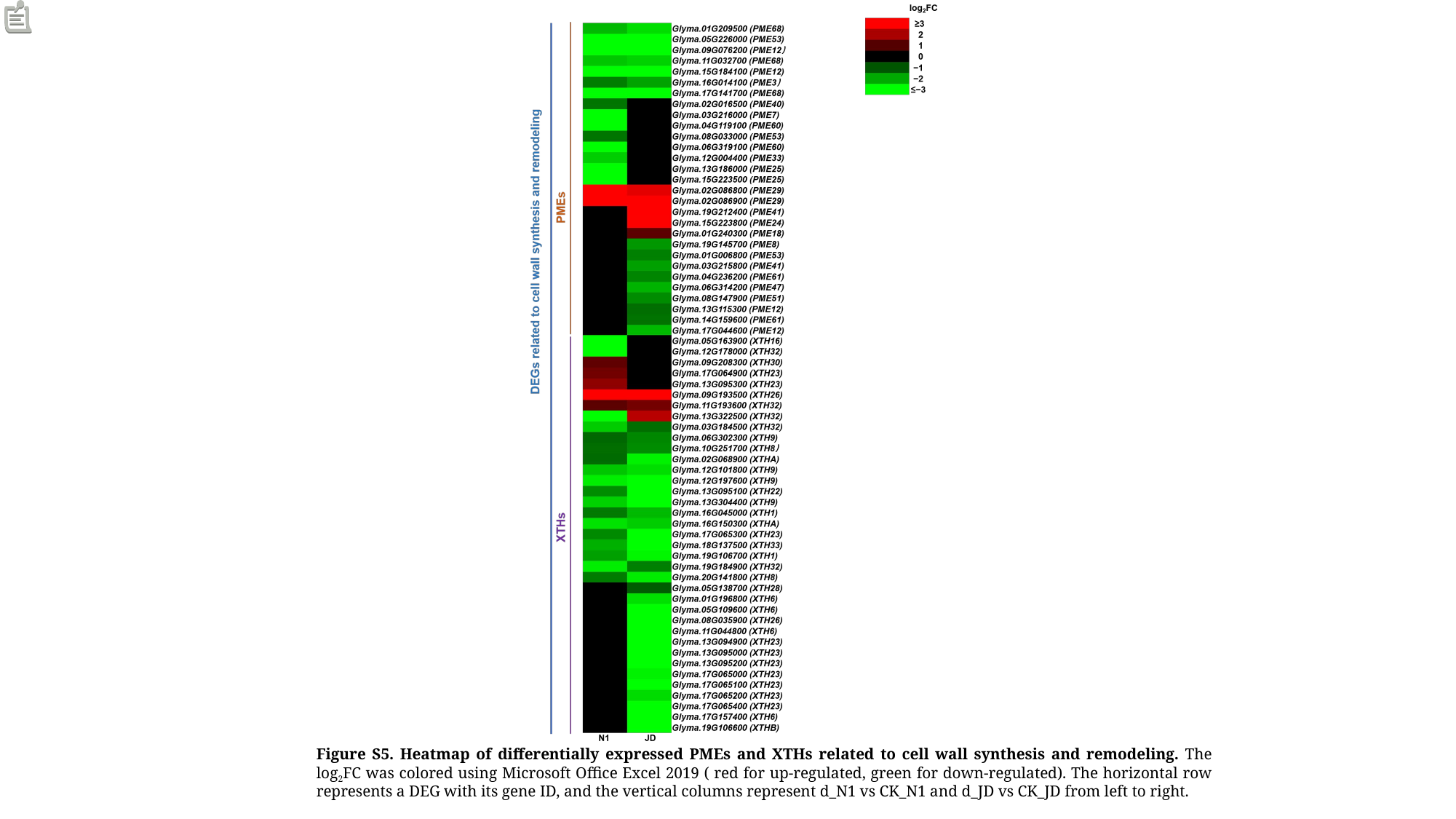

Figure S5. Heatmap of differentially expressed PMEs and XTHs related to cell wall synthesis and remodeling. The log2FC was colored using Microsoft Office Excel 2019 ( red for up-regulated, green for down-regulated). The horizontal row represents a DEG with its gene ID, and the vertical columns represent d_N1 vs CK_N1 and d_JD vs CK_JD from left to right.

## Slide 7
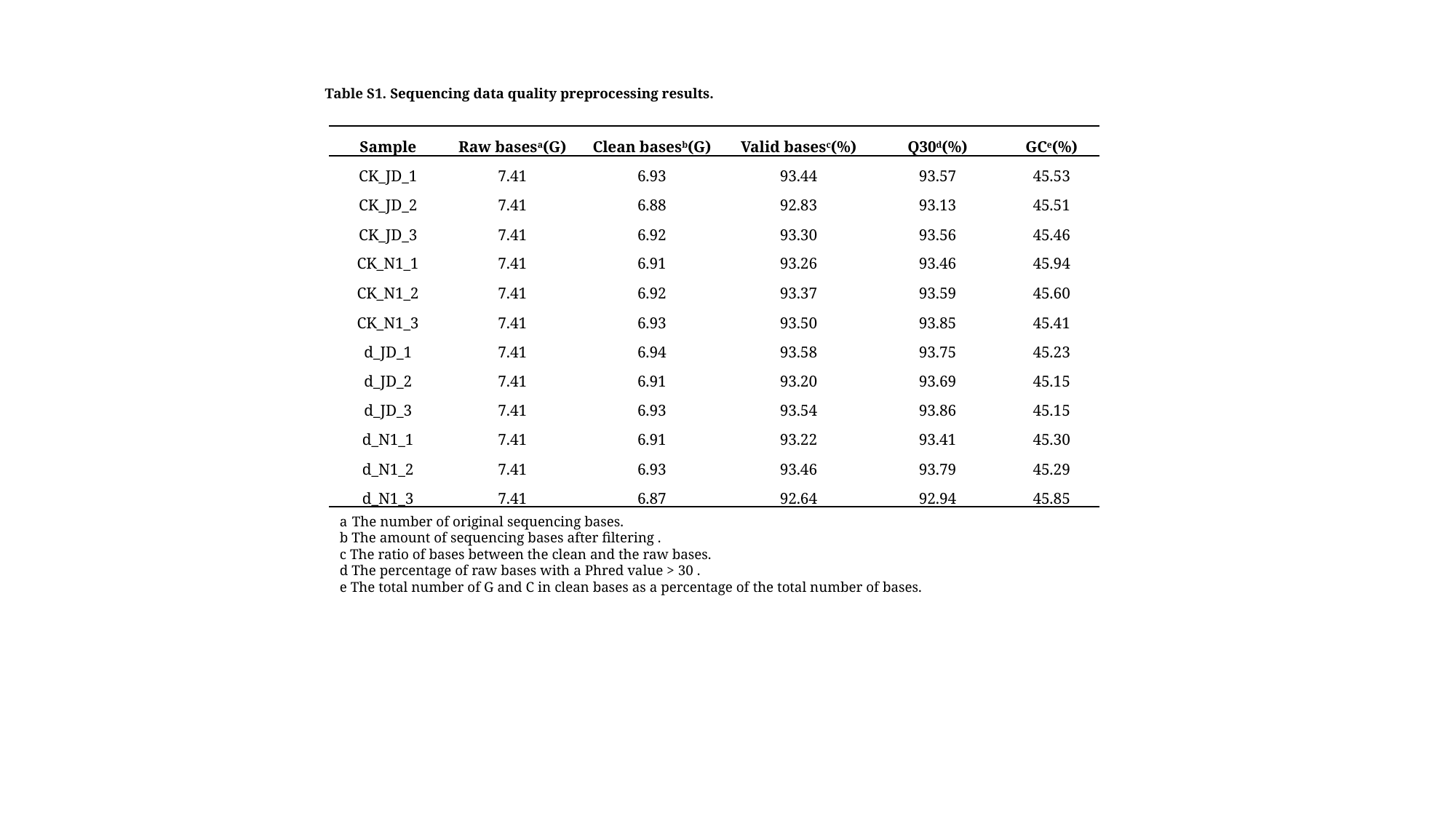

Table S1. Sequencing data quality preprocessing results.
| Sample | Raw basesa(G) | Clean basesb(G) | Valid basesc(%) | Q30d(%) | GCe(%) |
| --- | --- | --- | --- | --- | --- |
| CK\_JD\_1 | 7.41 | 6.93 | 93.44 | 93.57 | 45.53 |
| CK\_JD\_2 | 7.41 | 6.88 | 92.83 | 93.13 | 45.51 |
| CK\_JD\_3 | 7.41 | 6.92 | 93.30 | 93.56 | 45.46 |
| CK\_N1\_1 | 7.41 | 6.91 | 93.26 | 93.46 | 45.94 |
| CK\_N1\_2 | 7.41 | 6.92 | 93.37 | 93.59 | 45.60 |
| CK\_N1\_3 | 7.41 | 6.93 | 93.50 | 93.85 | 45.41 |
| d\_JD\_1 | 7.41 | 6.94 | 93.58 | 93.75 | 45.23 |
| d\_JD\_2 | 7.41 | 6.91 | 93.20 | 93.69 | 45.15 |
| d\_JD\_3 | 7.41 | 6.93 | 93.54 | 93.86 | 45.15 |
| d\_N1\_1 | 7.41 | 6.91 | 93.22 | 93.41 | 45.30 |
| d\_N1\_2 | 7.41 | 6.93 | 93.46 | 93.79 | 45.29 |
| d\_N1\_3 | 7.41 | 6.87 | 92.64 | 92.94 | 45.85 |
a The number of original sequencing bases.
b The amount of sequencing bases after filtering .
c The ratio of bases between the clean and the raw bases.
d The percentage of raw bases with a Phred value > 30 .
e The total number of G and C in clean bases as a percentage of the total number of bases.

## Slide 8
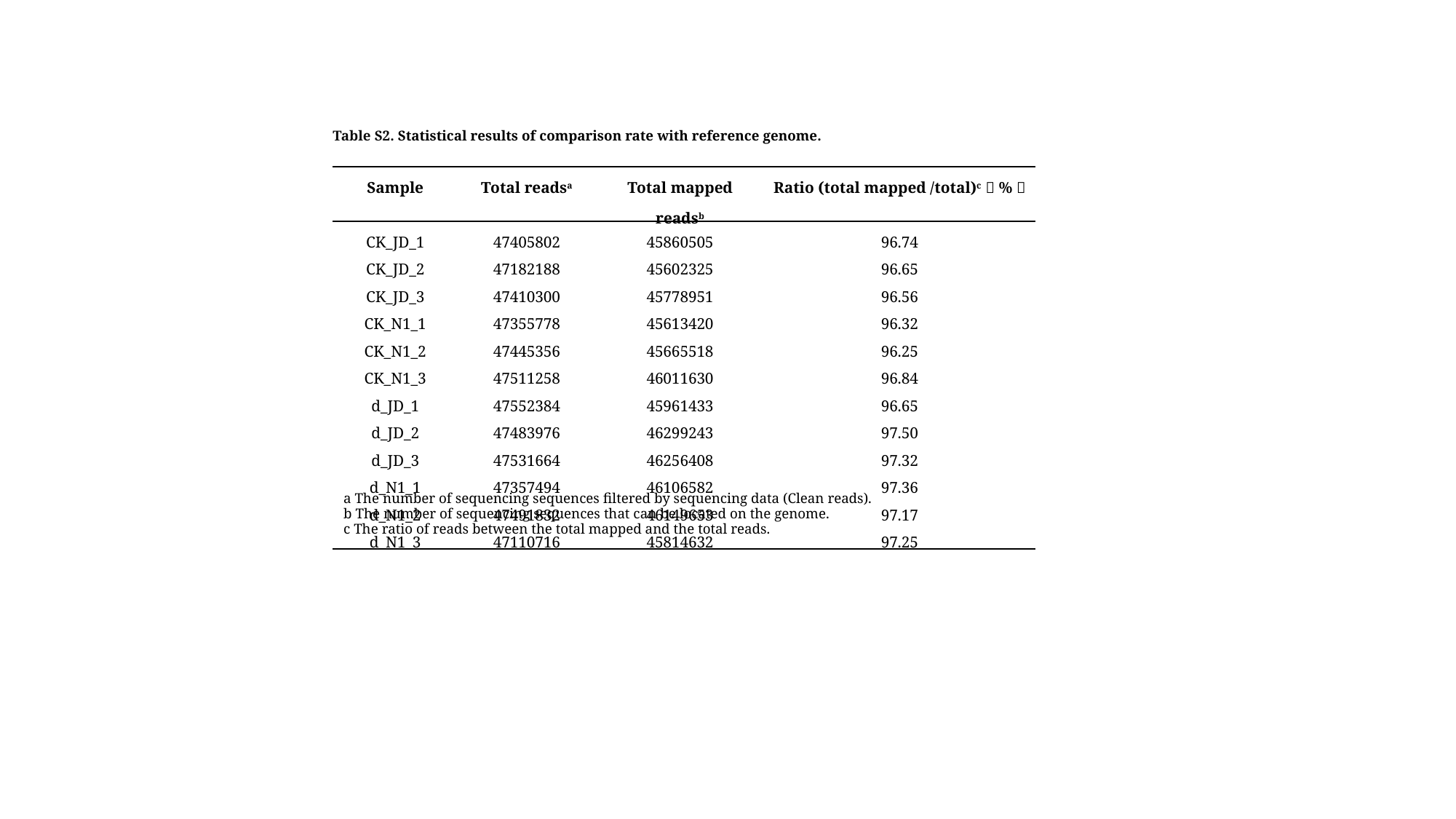

Table S2. Statistical results of comparison rate with reference genome.
| Sample | Total readsa | Total mapped readsb | Ratio (total mapped /total)c （%） |
| --- | --- | --- | --- |
| CK\_JD\_1 | 47405802 | 45860505 | 96.74 |
| CK\_JD\_2 | 47182188 | 45602325 | 96.65 |
| CK\_JD\_3 | 47410300 | 45778951 | 96.56 |
| CK\_N1\_1 | 47355778 | 45613420 | 96.32 |
| CK\_N1\_2 | 47445356 | 45665518 | 96.25 |
| CK\_N1\_3 | 47511258 | 46011630 | 96.84 |
| d\_JD\_1 | 47552384 | 45961433 | 96.65 |
| d\_JD\_2 | 47483976 | 46299243 | 97.50 |
| d\_JD\_3 | 47531664 | 46256408 | 97.32 |
| d\_N1\_1 | 47357494 | 46106582 | 97.36 |
| d\_N1\_2 | 47491832 | 46149653 | 97.17 |
| d\_N1\_3 | 47110716 | 45814632 | 97.25 |
a The number of sequencing sequences filtered by sequencing data (Clean reads).
b The number of sequencing sequences that can be located on the genome.
c The ratio of reads between the total mapped and the total reads.

## Slide 9
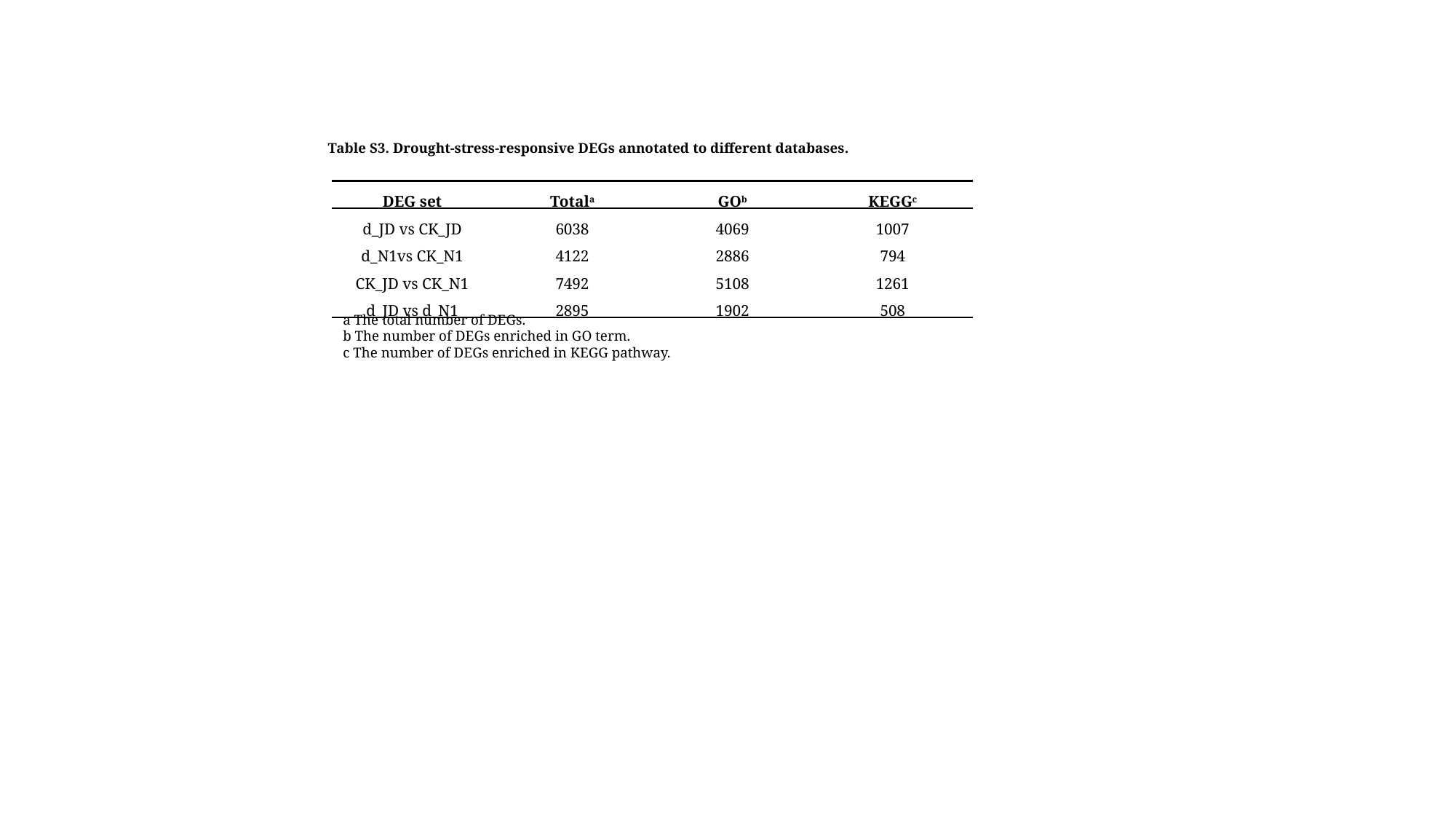

Table S3. Drought-stress-responsive DEGs annotated to different databases.
| DEG set | Totala | GOb | KEGGc |
| --- | --- | --- | --- |
| d\_JD vs CK\_JD | 6038 | 4069 | 1007 |
| d\_N1vs CK\_N1 | 4122 | 2886 | 794 |
| CK\_JD vs CK\_N1 | 7492 | 5108 | 1261 |
| d\_JD vs d\_N1 | 2895 | 1902 | 508 |
a The total number of DEGs.
b The number of DEGs enriched in GO term.
c The number of DEGs enriched in KEGG pathway.

## Slide 10
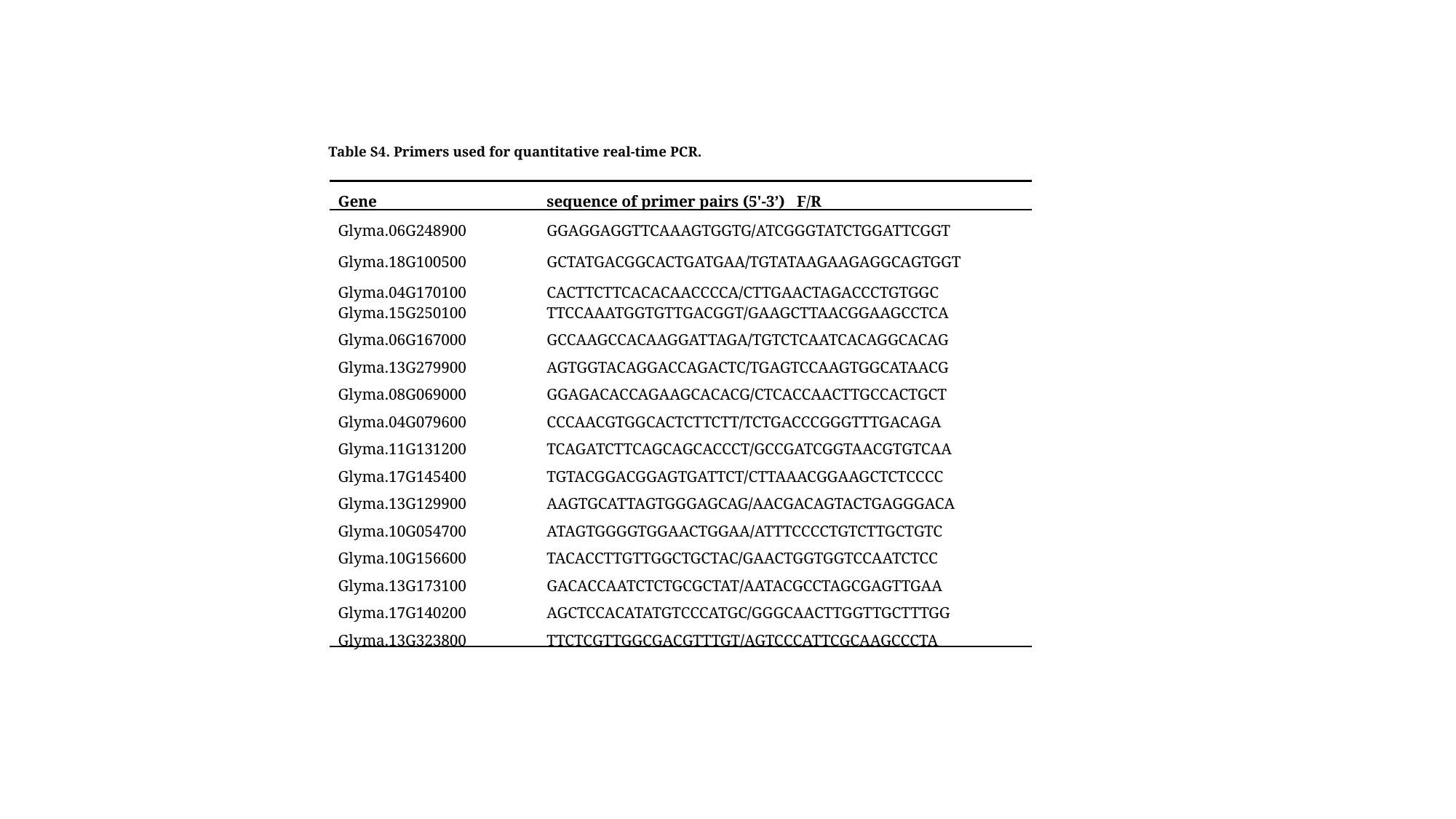

Table S4. Primers used for quantitative real-time PCR.
| Gene | sequence of primer pairs (5'-3’) F/R |
| --- | --- |
| Glyma.06G248900 Glyma.18G100500 Glyma.04G170100 | GGAGGAGGTTCAAAGTGGTG/ATCGGGTATCTGGATTCGGT GCTATGACGGCACTGATGAA/TGTATAAGAAGAGGCAGTGGT CACTTCTTCACACAACCCCA/CTTGAACTAGACCCTGTGGC |
| Glyma.15G250100 | TTCCAAATGGTGTTGACGGT/GAAGCTTAACGGAAGCCTCA |
| Glyma.06G167000 | GCCAAGCCACAAGGATTAGA/TGTCTCAATCACAGGCACAG |
| Glyma.13G279900 | AGTGGTACAGGACCAGACTC/TGAGTCCAAGTGGCATAACG |
| Glyma.08G069000 | GGAGACACCAGAAGCACACG/CTCACCAACTTGCCACTGCT |
| Glyma.04G079600 | CCCAACGTGGCACTCTTCTT/TCTGACCCGGGTTTGACAGA |
| Glyma.11G131200 | TCAGATCTTCAGCAGCACCCT/GCCGATCGGTAACGTGTCAA |
| Glyma.17G145400 | TGTACGGACGGAGTGATTCT/CTTAAACGGAAGCTCTCCCC |
| Glyma.13G129900 | AAGTGCATTAGTGGGAGCAG/AACGACAGTACTGAGGGACA |
| Glyma.10G054700 | ATAGTGGGGTGGAACTGGAA/ATTTCCCCTGTCTTGCTGTC |
| Glyma.10G156600 | TACACCTTGTTGGCTGCTAC/GAACTGGTGGTCCAATCTCC |
| Glyma.13G173100 | GACACCAATCTCTGCGCTAT/AATACGCCTAGCGAGTTGAA |
| Glyma.17G140200 | AGCTCCACATATGTCCCATGC/GGGCAACTTGGTTGCTTTGG |
| Glyma.13G323800 | TTCTCGTTGGCGACGTTTGT/AGTCCCATTCGCAAGCCCTA |
